# Varicella Zoster Virus and Large Vessel Vasculitis, the Absence of an Association

**DOI:** 10.20411/pai.v2i2.196

**Published:** 2017-06-06

**Authors:** Gary W. Procop, Charis Eng, Alison Clifford, Alexandra Villa-Forte, Leonard H. Calabrese, Eric Roselli, Lars Svensson, Douglas Johnston, Gosta Pettersson, Edward Soltesz, Lisa Lystad, Julian D. Perry, Alexander Blandford, Deborah A. Wilson, Gary S. Hoffman

**Affiliations:** 1 Pathology and Laboratory Medicine Institute, Cleveland Clinic, Cleveland, Ohio; 2 Genomic Medicine Institute, Lerner Research Institute, Taussig Cancer Institute, Cleveland Clinic, Cleveland, Ohio; 3 Center for Vasculitis Care and Research, Department of Rheumatic and Immunologic Diseases, Cleveland Clinic, Cleveland, Ohio; 4 Division of Rheumatology, University of Alberta, Canada; 5 Heart & Vascular Institute, Cleveland Clinic, Cleveland, Ohio; 6 Cole Eye Institute, Cleveland Clinic, Cleveland, Ohio

**Keywords:** Aorta and temporal artery biopsies, Varicella Zoster Virus, Large Vessel Vasculitis

## Abstract

**Objective:**

It is controversial whether microorganisms play a role in the pathogenesis of large and medium vessel vasculitides (eg, giant cell arteritis [GCA], Takayasu arteritis [TAK] and focal idiopathic aortitis [FIA]). Recent studies have reported the presence of Varicella Zoster Virus (VZV) within formalin-fixed, paraffin-embedded temporal arteries and aortas of about three-quarters or more of patients with these conditions, and in a minority of controls. In a prospective study, we sought to confirm these findings using DNA extracted from vessels that were harvested under surgically aseptic conditions and snap frozen.

**Methods and Results:**

DNA samples extracted from 11 surgically sterile temporal arteries and 31 surgically sterile thoracic aortas were used in an attempt to identify the vessel-associated VZV genome. Two different validated PCR methods were used. Thirty-one thoracic aorta aneurysm specimens included biopsies from 8 patients with GCA, 2 from patients with TAK, 6 from patients with FIA, and 15 from patients without vasculitis, who had non-inflammatory aneurysms. Eleven temporal artery biopsies were collected from 5 patients with GCA and 6 controls. The presence of VZV was not identified in either the specimens from patients with large vessel vasculitis or from the controls.

**Conclusions:**

Using surgically sterile snap-frozen specimens, we were unable to confirm recent reports of the presence of VZV in either aortas or temporal arteries from patients with large vessel vasculitis or controls.

## INTRODUCTION

Within the broad spectrum of vasculitis, infection has been proven to play a role in a minority of cases. The best known associations are with hepatitis B and C, HIV, syphilis, and tuberculosis. Numerous other bacteria, viruses, and fungi have sporadically been implicated as etiologic agents of vasculitis [1]. In a minority of patients with isolated central nervous system vasculitis or vasculopathy, VZV appears to have been the inciting agent for vascular injury. Over the past several years, a series of studies using immunohistochemistry (IHC) and PCR performed on formalin-fixed, paraffin-embedded (FFPE) temporal arteries and aortas, have noted the presence of VZV antigen or nucleic acid in the vessel walls of a majority of 163 GCA cases and in 11 cases of aortitis. A minority of non-inflammatory temporal artery and aorta controls were also VZV-positive by IHC and PCR [2–5].

Because these observations have profound implications for understanding and treating large vessel vasculitis (LVV), we felt it was critical to test the reproducibility of these findings in tissue samples collected under more ideal surgically sterile and not embedded conditions.

## PATIENTS AND METHODS

Our study included patients undergoing thoracic aorta reconstruction for aneurysms caused by LVV and non-inflammatory conditions such as hypertension or congenital/genetic anomalies (eg, Bicuspid aortic valve, Marfan, or Loeys-Dietz syndrome) that served as controls for LVV. Temporal artery biopsies, not including skin, were obtained from patients suspected of having GCA.

All specimens were obtained and maintained in a surgically sterile manner. Biopsies were split, with one half sent for routine histopathological review and the other half snap-frozen in liquid nitrogen and maintained at -80°C until analysis. Patients with GCA who had undergone temporal artery biopsy, were classified according to their clinical phenotype and histopathological findings as either biopsy-positive (histopathology confirming inflammatory infiltrates and compatible clinical presentation) or biopsy-negative GCA (histopathology showing absence of inflammatory infiltrates, but meeting the American College of Rheumatology 1990 Classification criteria for GCA [6] and with a persistent clinical diagnosis of GCA at 3 months post-biopsy). Controls were patients in whom the diagnosis of GCA was subsequently excluded (based on histopathology and clinical results.) We have defined FIA as a condition not associated with clinically apparent systemic vasculitis or other autoimmune diseases, and in which no evidence of vasculitis exists beyond the surgical site at the time of diagnosis [7–9].

Deoxyribonucleic acid (DNA) isolation: Total DNA was isolated using a modified version of a previously published protocol [10]. In brief, modifications included bead homogenization of fresh tissue at 30 Hz for 60 seconds in TissueLyser II (Qiagen, Valencia, CA), with a hybrid non-enzymatic yeast and bacterial cell wall lysis (Epicentre, Madison, WI) following proteinase K (Roche Diagnostics Corporation, Indianapolis, IN) digestion. Finally, DNA was isolated via standard ethanol precipitation.

We used 2 rapid-cycle PCR assays for the detection of VZV in the tissue DNA extracts. These assays consisted of a LightCycler assay and a commercially available VZV PCR kit (RealStar® VZV PCR Kit, Altona Diagnostics, Hamburg, Germany). The details of these assays have been previously described [11]. In brief, the first VZV-specific PCR assay was performed on the Roche LightCycler 1.2 system. The primers and fluorescence-resonance energy transfer probes used with this assay were as follows: forward primer, 5′-GAC AAT ATC ATATACATG GAATGT G-3′; reverse primer, 5′-GCG GTA GTA ACAGAG AAT TTC TT-3′; hybridization probe-1, 5′-CGA AAA TCCAGAATCGGAACT TCT T-FITC-3′; and hybridization probe-2, 5′-LC640-CCA TTA CAG TAA ACT TTA GGC GGT C-3′. The LightCycler PCR protocol consisted of 10 minutes at 95°C for *Taq* polymerase activation, 45 cycles of PCR amplification (95°C for 10 seconds, 60°C for 10 seconds, and 72°C for 20 seconds), a melting step (40°C to 95°C at 0.1°C/s), and a cooling step (40°C for 30 seconds).

The RealStar VZV PCR kit (Altona Diagnostics, Hamburg, Germany) was also used to detect VZV by rapid-cycle PCR. This assay was performed on the Rotor-Gene^®^ 6000 (QIAGEN^®^, Germany). The PCR protocol consisted of 10 minutes at 95°C and 45 cycles of amplification (95°C for 15 seconds, 58°C for 1minute). The amplified product was detected in the FAM (6-carboxyfluorescein) channel and the internal control included with this assay was detected in the JOE (5′-dichloro-dimethoxy-fluorescein) channel. The sequences of the primers and probe used in this assay are proprietary. The analytical sensitivity of the RealStar VZV assay is 0.1 copies/μL (95% CI: 0.05–0.3 copies/μL). We made dilutions of the extract from the positive control.

Positive controls for both VZV PCR assays were nucleic acid extracts from a vesicular skin (neck) lesion, which was caused by VZV as demonstrated by both direct immunofluorescence (DFA) and viral culture. The skin biopsy with a vesicle was diluted using serial 1:10 dilutions. Dilutions were PCR positive from 1.0 x 10^-1^ through 1.0 x 10^-4^ dilutions and became PCR negative at 1.0 x 10^-5^ dilution. Viral quantification was uncertain because the starting concentration of virus in the positive control was unknown. This positive external amplification control was used primarily to assure the validity of the PCR mastermix (ie the desired target was appropriately amplified).

The study (IRB#10-1128) was approved by the Institutional Review Board of the Cleveland Clinic.

## RESULTS

Among patients undergoing surgery for proximal aortic aneurysms or diagnostic temporal artery biopsies, 31 thoracic aorta and 11 temporal artery biopsy specimens were examined (Table 1). Among patients with GCA, 8 with aorta biopsies had a mean age of 70 (range 63–81) years and 5 with temporal artery biopsies had a mean age of 74 (70–81) years. All GCA patients were white and 85.0% were female. Among 6 control patients undergoing temporal artery biopsy, the mean age was 70 (51–87) years, 83% were female, and all but one who declined to state her race were white. Two patients undergoing aorta surgery had Takayasu arteritis, 1 male and 1 female, aged 30 and 39 years. Among 6 patients with FIA, the mean age was 63 (range 45–82) years, 4 were female, 1 Asian, 1 African American, and 4 white. The 15 patients who were controls with non-inflammatory aorta biopsies had a mean age of 58 years and a wide age range (25–78), reflecting the demographics of congenital vascular anomalies (eg bicuspid aortic valve-associated aneurysms) and chronic degenerative aortic disease (eg hypertension) associated with cystic medial degeneration. Sixty percent were female and all were white. None of the control patients having aorta aneurysm surgery were receiving corticosteroids, whereas 3 of 8 patients with GCA having aorta surgery were receiving 2, 5, and 35 mg/day of prednisone. All patients undergoing temporal artery biopsies, those with GCA and controls, were receiving corticosteroids.

**Table 1:** Aorta and Temporal Artery Biopsies. Diagnostic Subsets and Comorbidities

Case number within subsets	Diagnosis	Age	Gender	Race	Comorbidities	Prednisone dose mg/d*
	**Inflammatory aortic disease**					
1	GCA	76	F	W	hypertension	2
2	GCA	81	F	W	Polymyalgia rheumatica, hypertension, hyperlipidemia A-fib	5
3	GCA	66	M	w	none	35
4	GCA	67	F	w	MV prolapse hypertension	0
5	GCA	71	F	w	Hypertension, CAD	0
6	GCA	70	F	w	CAD	0
7	GCA	65	F	w	hyperlipidemia	0
8	GCA	63	F	w	CAD hypertension	0
1	TAK	30	M	w	none	20
2	TAK	39	F	w	none	30
1	FIA	67	M	w	Hypertension hyperlipidemia	0
2	FIA	70	F	C	CAD, CHF, A-fib, hypertension	0
3	FIA	53	M	Asian	none	0
4	FIA	62	F	W	Bicuspid Aortic, Valve, CAD Hypertension Hyperlipidemia	0
5	FIA	45	F	W	Hyperlipidemia	0
6	FIA	82	F	AA	A-fib, hypertension, Type 2 diabetes	0

Aortic aneurysm non-inflammatory Controls						
1		75	F	W	hypertension	0
2		57	F	W	Bicuspid AV	0
3		66	M	W	CAD, hypertension, hyperlipidemia	0
4		69	F	W	none	0
5		76	F	W	Hypertension, hyperlipidemia	0
6		57	F	W	none	0
7		25	M	W	Bicuspid AV	0
8		32	M	W	Bicuspid AV	0
9		78	M	W	none	
10		65	F	W	Hypertension, hyperlipidemia, A-fib	0
11		51	M	W	Bicuspid AV	0
12		55	F	W	Hypertension, Type 2 DM, hyperlipidemia, CAD	0
13		50	F	W	Bicuspid AV	0
14		68	M	W	none	0
15		47	F	W	Bicuspid AV	0

Temporal artery biopsy	Diagnosis	Age	Gender	Race	Comorbidities	Prednisone-Dose @ bx (mg/d)**
1	GCA	70	M	W	Past history of PMR, ascending aortic aneurysm, stroke, kidney stones, obstructive sleep apnea	40
2	GCA	70	M	W	CABG x2, osteogenesis imperfecta, fractures, hearing loss, acute VL both eyes	1000
3	GCA	77	F	W	Left eye vision loss since birth, GERD, hypertension, osteoarthritis	30
4	GCA	70	F	W	osteoarthritis, migraine, carpal tunnel, sarcoidosis (lymph node Bx +1977)—resolved, MGUS, peripheral neuropathy	40
5	GCA	81	F	W	Prior history of breast CA, colon cancer and TIA	10
1	Control	51	F	W	DVT, stroke, antiphospholipid antibody syndrome, migraine, depression, Hashimoto thyroiditis	40
2	Control	76	F	Patient declined to state race	Basal cell carcinoma, COPD, CHF, depression, hypothyroidism, chronic migraine, hypertension, stroke, seizures	80
3	Control	59	F	W	Migraine, sensori-neural hearing loss, hypothyroidism	60
4	Control	63	F	W	GERD, asthma, depression, morphea, spinal stenosis, osteoarthritis irritable bowel syndrome, peptic ulcer disease	60
5	Control	87	F	W	RA, A. fib, aortic stenosis, sensorineural hearing loss, osteoporosis, COPD hyperparathyroidism	60
6	Control	83	M	W	Diverticulitis, GERD, obstructive sleep apnea, AAA (3.7 cm)	40

*None of the control aorta patients were receiving corticosteroids or other immunosuppressive agents None of the aortitis patients were receiving corticosteroids *plus* another immunosuppressive agent.

**None of the GCA or temporal artery control patients were receiving corticosteroids *plus* another immunosuppressive agent.

**Figure 1.**
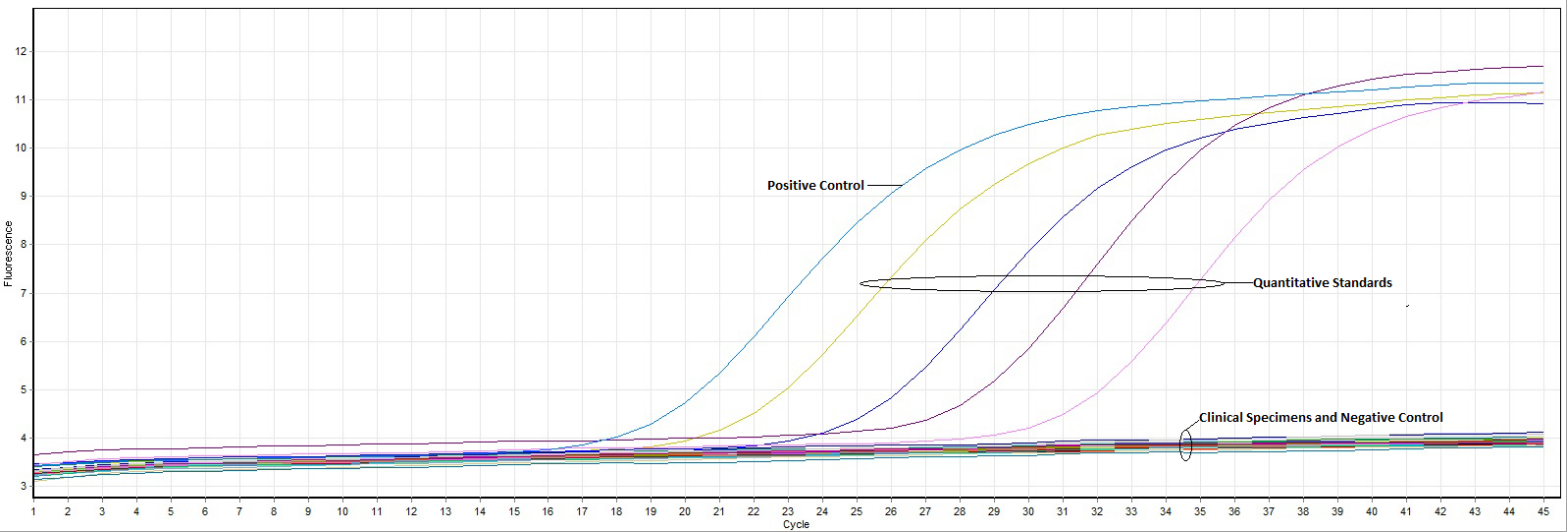
RealStar VZV PCR kit results demonstrate the absence of VZV in biopsies from patients with aortitis, giant cell arteritis, and clinical controls. The positive and negative external controls have amplified appropriately. Additionally, the quantitative amplification controls, which are external quantitative standards supplied by the manufacturer, have reacted appropriately; these have the following concentrations (left to right): 1.0 x 10^4^ copies/μL, 1.0 x 10^3^ copies/μL, 1.0 x 10^2^ copies/μL, and 1.0 x 10^1^ copies/μL.

The concentration of DNA extracted from biopsies varied from 1.97–72.12 ng/uL of tissue and the volume of tissue varied from 6–40 uL. Using both the LightCycler and Altona VZV kits, all specimens with vasculitis as well as biopsies with negative results were VZV negative. The internal control included in the Astra VZV kit was amplified in each of the samples tested, indicating that our negative results for cases and controls were not false negatives (Figure 1).

## DISCUSSION

To date, this is the only study to use thoracic aorta and temporal artery biopsy specimens, which were prepared in a surgically sterile manner and snap-frozen, in an effort to identify VZV in LVV and controls. Prior studies that used formalin-fixed tissue may have introduced errors because formalin is known to cause DNA cross-linking, nucleic acid shearing, and sequencing artifacts [12]. While this is a methodological limitation, it would be expected to under-estimate the yield of VZV and is unlikely to introduce VZV into vascular tissue. Our freshly frozen temporal artery samples, were collected in an aseptic fashion and specifically for these and ongoing vascular microbiome studies. Such specimens are best suited to explore the actual microbial constituents in tissue. However, rather than confirm prior reports of VZV in LVV, we were unable identify VZV in any of our vasculitis or control specimens.

That certain forms of vasculitis may be due to infection is an attractive idea. Indeed, in a minority of cases, vasculitis has been associated with numerous infectious agents. This topic has recently been reviewed with regard to bacteria and viruses within diseased blood vessels [1]. Evidence for infection enhancing vascular injury is most compelling in atherosclerosis. Recent reports have also raised questions about the potential role of microbes in non-atherosclerotic aortic aneurysms and vasculitis [13]. Preliminary evidence also suggests that apparently normal vessels may harbor microbes. With the exception of certain viral infections (eg, hepatitis C virus, HIV, Epstein-Barr virus, and cytomegalovirus) and infectious endocarditis, systemic vasculitides have not been convincingly associated with infectious agents.

VZV is a ubiquitous agent. Over 95% of adults have serologic evidence of prior infection and are presumed to have latent VZV that may reactivate in over 50% of people by the age of 85. In addition, VZV has been associated with vasculopathies including ischemic and hemorrhagic stroke, cranial neuropathy, and spinal cord infarction, making it an attractive candidate for causing certain forms of vasculitis presumed to be idiopathic and autoimmune [2]. Studies by Gilden, Nagel, and colleagues using immunohistochemistry (IHC) techniques and PCR of FFPE samples of temporal arteries and aortas have noted the presence of VZV antigen in about 3/4 of cases of GCA by IHC and in all of 11 evaluated cases of aortitis. Forty percent of GCA cases with amplifiable DNA had VZV-positive results by PCR [3–5]. Whether or not the diagnosis of GCA was based on clinical findings, with positive or negative results for temporal artery biopsies, 73% of 93 biopsies and 64% of 70 biopsies, respectively, were VZV-IHC positive. Twenty-two percent of asymptomatic controls (11/49), without a history of vasculitis, and with biopsies devoid of inflammation also had positive results by IHC. Eleven control temporal artery biopsies with normal results demonstrated VZV antigen by IHC, and 3 of 9 with amplifiable DNA contained VZV DNA [3].

In a separate study by the Gilden lab, 29 FFPE proximal aorta specimens were studied. Eleven of 29 specimens had granulomatous aortitis (5 GCA or TAK and 6 idiopathic aortitis) and all tested positive for VZV by IHC. Among the 18 non-inflammatory aorta controls, 5 (28%) were also VZV IHC-positive and 4 of 5 (22% of 18 aortitis cases) had aorta wall in which VZV was detected by PCR [5]. The authors suggested that positive controls in each of these studies indicated that in addition to age and VZV, unknown co-factors are required to develop GCA, TAK, or aortitis. They concluded that in most patients with GCA, VZV may trigger disease and that anti-viral therapy may be of benefit. These observations have not been supported by others who also used PCR to study FFPE biopsies [14–18].

Because the positive observations implicating VZV in LVV have profound implications for understanding and treating LVV, we felt it was critical to test the reproducibility of these findings by using tissue samples collected under more ideal conditions. Our inability to replicate these results was disappointing and did not provide an explanation for differences between our findings.

The limitations of our study include having studied only 42 biopsies (31 thoracic aorta and 11 temporal artery) for which there were varying concentrations of extracted DNA. Nonetheless, power calculations show that a sample size of 40 is sufficiently powered (*P* > 0.9) even if the prevalence of VZV were only 5%. Furthermore, amplification of the internal control excluded false negative results. Additionally, the positive amplification controls functioned as expected.

The strengths of our study are in the use of 2 validated PCR kits, incorporating probes for VZV and use of surgically sterile snap-frozen and non-embedded biopsies from patients with LVV and non-inflammatory vasculopathy controls. This approach is least likely to produce artifacts. Our study was limited to searching for VZV and does not rule out the presence of other microbial agents playing a role in vessel health and diseases such as in the vasculitides

## CONCLUSION

Our study, using surgically sterile biopsies, does not support the finding of VZV being an etiologic agent in large vessel vasculitides (GCA, TAK, focal idiopathic aortitis). Conflicting data in the literature should be a caution to clinicians who consider the use of anti-VZV therapies in large vessel vasculitis.
